# Concomitant versus Delayed Cholecystectomy in Bariatric Surgery

**DOI:** 10.1155/2021/9957834

**Published:** 2021-06-14

**Authors:** Hatem Elgohary, Mahmoud El Azawy, Mohey Elbanna, Hossam Elhossainy, Wael Omar

**Affiliations:** ^1^General Surgery Department, Faculty of Medicine, Helwan University, Helwan, Egypt; ^2^Department of General Surgery, Faculty of Medicine, Ain-shams University, Cairo, Egypt

## Abstract

**Background:**

Obesity and weight loss after bariatric surgery have a close association with gallbladder disease. The performance and proper timing of laparoscopic cholecystectomy (LC) with bariatric surgery remain a clinical question.

**Objective:**

Evaluation of the outcome of LC during bariatric surgery whether done concomitantly or delayed according to the level of intraoperative difficulty.

**Methods:**

The prospective study included patients with morbid obesity between December 2018 and December 2019 with preoperatively detected gallbladder stones. According to the level of difficulty, patients were allocated into 2 groups: group 1 included patients who underwent concomitant LC during bariatric surgery, and group 2 included patients who underwent delayed LC after 2 months. In group 1, patients were further divided into subgroups: LC either at the beginning (subgroup A) or after bariatric surgery (subgroup B).

**Results:**

Operative time in group 1 vs. 2 was 92.63 ± 28.25 vs. 68.33 ± 17.49 (*p* < 0.001), and in subgroup A vs. B, it was 84.19 ± 19.62 vs. 130.0 ± 31.62 (*p* < 0.001). One patient in each group (2.6% and 8.3%) had obstructive jaundice, *p* > 0.001. In group 2, 33% of asymptomatic patients became symptomatic for biliary colic *p* > 0.001. LC difficulty score was 2.11 ± 0.70 vs. 5.66 ± 0.98 in groups 1 and 2, respectively, *p* < 0.001. LC difficulty score decreased in group 2 from 5.66 ± 0.98 to 2.26 ± 0.78 after 2 months of bariatric surgery, *p* < 0.001.

**Conclusion:**

Timing for LC during bariatric surgery is challenging and should be optimized for each patient as scheduling difficult LC to be performed after 2 months may be an option.

## 1. Introduction

The presence of asymptomatic gallstones (GS) is no longer an indication for elective prophylactic laparoscopic cholecystectomy (LC) according to the recommendations of the 1991 French Consensus Conference on cholelithiasis. However, there may be potential benefits of performing prophylactic LC during certain abdominal procedures for nonbiliary disease; this remains a subject of debate. This debate has become livelier with the recent increase in bariatric surgery (BS) [1].

The debate for the optimal timing of LC arises from the fact that BS leads to a higher risk for gallbladder pathologies due to the massive weight reduction, and it is also associated with altered gastrointestinal anatomy. On the other hand, adding concomitant LC to a BS poses certain risks due to the increased morbidity for patients with obesity, and it may be technically challenging due to excess intra-abdominal fat and difference in ports' placement for BS and LC with difficulties establishing and maintaining pneumoperitoneum, the higher incidence for conversion to open surgery and bile duct injuries [2, 3].

The time interval allows LC to be performed more safely due to loss of intra-abdominal fat and the possible late complications of BS; for example, port site hernia can be operated on simultaneously [4]. Therefore, the surgeon has to weigh the potential added risk of concomitant LC with the potential morbidity of delayed complications from GS [5].

Concomitant cholecystectomy for asymptomatic cholelithiasis during a planned abdominal operation is a common clinical scenario as several studies showed a high (up to 70%) incidence of symptoms and/or complications from the biliary system (such as biliary colic, acute cholecystitis, and jaundice) in patients with asymptomatic cholelithiasis following laparotomy for unrelated conditions, and cholecystectomy was required in a large percentage (up to 40%) of these patients within 1 year of the initial operation [6].

Unlike laparoscopic roux-en-y gastric bypass (LRYGB), laparoscopic sleeve gastrectomy (LSG) does not include any bypassed intestinal segment and, therefore, does not affect the enterohepatic circulation, and endoscopic treatment of bile duct stones would be easier when needed. Moreover, serum bile acid levels are elevating within days following LRYGB, when compared preoperatively and when compared to weight-matched nonsurgical individuals. On the contrary, Belgaumkar et al. reported that serum bile acid levels after LSG did not significantly change. As a result, the management of asymptomatic GS for LSG and LRYGB should be different [4, 7].

This present study aims to evaluate the outcome of LC during BS whether done concomitantly or delayed according to the level of intraoperative difficulty.

## 2. Patients and Methods

This is a prospective study conducted in Helwan University Hospitals in Egypt including 50 patients with morbid obesity who had been diagnosed with GS by preoperative abdominal ultrasound (US) and underwent BS and were consecutively selected during the period between December 2018 and December 2019.

Eligibility for BS was defined according to BMI ≥35 kg/m^2^ with one or more obesity-related comorbidities and a BMI >40 kg/m^2^ without coexisting comorbid conditions and to whom BS would not pose an excessive risk [8].

Patients were divided into two main groups according to the level of intraoperative difficulty of LC using the intraoperative difficulty score (Table 1) [2]. We depended on inspection and examination by noncrushing graspers for gallbladder and Calot's triangle. Group 1 included patients with a score <5 and they underwent concomitant LC, and group 2 included patients with a score ≥5 and they underwent delayed LC after 2 months of BS. It was the time of maximal weight loss, and patients began to eat well at 2 months to avoid complications of the effect of rapid weight loss on gall stones.

Moreover, patients in group 1 were randomly subdivided by the head surgeon using the blocking method of randomization into two subgroups: subgroup A included patients who underwent LC before the beginning of BS, and subgroup B included patients who underwent LC after the completion of BS at the same session.

Preoperative factors such as sex, BMI, diabetes mellitus, previous surgery, history of cholecystitis, white blood cell count, GB wall thickness, and presence of pericholecystic fluid had all been demonstrated to be predictive factors for surgical difficulty [9].

Group 2 patients were screened postoperatively for symptoms or complications attributed to GS, and the diagnosis was achieved through clinical examination, blood work (complete blood picture and liver function tests), and the abdominal US.

An informed consent form was taken from all patients who were invited to participate in this study, and ethical committee approval was obtained.

Patients were excluded from the study if they had Mirizzi syndrome (type 1 or 2), a history of calcular obstructive jaundice, and a history of upper abdominal surgery.

All patients were subjected to the following.

### 2.1. Preoperative Assessment

Patients' demographics, anthropometric features, years of obesity, and comorbidities were recorded. Preoperative laboratory investigations were complete blood cell count, kidney, liver, and bleeding profile, and thyroid and cortisol panels. For cardiopulmonary workup, a routine chest radiograph is performed on all patients. Those at high risk for pulmonary complications received a sleep study and pulmonary function tests. Routine abdominal ultrasound was performed on all patients to detect GB pathology and the size of the liver.

### 2.2. Surgical Technique

All the operations were done by the same surgeons following the same surgical technique for both BS and LC. All procedures were performed under general anesthesia with endotracheal intubation in the French position, and prophylactic antibiotic (ceftriaxone 1 gm vial) was given intravenously at the induction of anesthesia.

We used five ports, and pneumoperitoneum was established through a 10 mm supraumbilical Visiport. The liver retractor was inserted through a 5 mm port just under the xiphoid process, and another 12 and 15 mm ports were placed on the left and right-middle clavicular lines, respectively, as a working port. Another 5 mm port was placed on the left anterior auxiliary line for the assistant.

#### 2.2.1. LSG

The dissection started on the greater curvature 4 cm from the pylorus up to the cardioesophageal junction until full mobilization of the gastric fundus. After dissecting the stomach from the greater curvature using LigaSure, a 36Fr calibration tube (bougie) was inserted into the stomach, and the resection was done by linear stapler starting 4 cm from the pylorus up to 1 cm away from the angle of Hiss.

#### 2.2.2. OAGB/MGB

The first step is the creation of a window in a lesser omentum below the level of the crow's foot. Then, the first horizontal firing was from the right port using an endo-GIA stapler 45 mm. Then, the vertical firings were from the left port after the introduction of a 36Fr calibration tube aiming to create a long gastric pouch. After that, a loop of small bowel approximately 200 cm from duodenojejunal flexure was then anastomosed to the gastric pouch in antecolic, retrogastric fashion. Stapler entry site was closed using 2/0 Vicryl in 2 layers. A leak test was performed using a dilute methylene blue solution in all cases, and an intra-abdominal drain was placed inside the peritoneal cavity.

#### 2.2.3. LC

**Figure 1:**
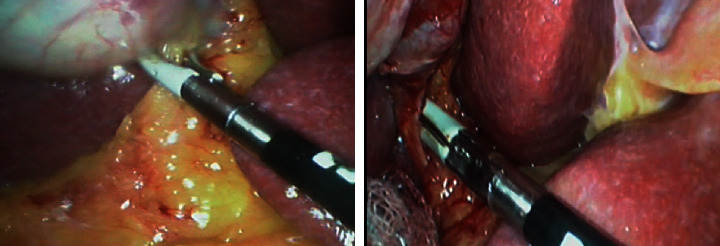
Laparoscopic cholecystectomy by LigaSure.

LC was performed using the right and left midclavicular ports as a working port. Initial dissection of Calot's triangle was followed by GB dissection off the liver bed using conventional hook or LigaSure (Covidien – USA). (Figure 1).

### 2.3. Postoperative Management

- A prophylactic dose of low molecular weight heparin started 12 hours postoperatively, and elastic stockings during the hospitalization were used for prophylaxis against thromboembolism. A clear liquid sip was started the second day of the operation, and intra-abdominal drains were removed after 24 hours if there is less than 50 ml serosanguinous fluid and it was left in place and removed at the first outpatient clinic (OPC) visit in patients with unusual operative bleeding, a higher risk for postoperative bleeding, and complex operative cases
- All patients without any complication were discharged on the second postoperative day after instructing on diet, activities, and medications including multivitamins
- In group 2: delayed LC was done after 2 months, and the intraoperative findings and postoperative outcomes were evaluated. And during this waiting period, all the patients were screened for biliary symptoms (biliary colic, cholecystitis, acute cholangitis, obstructive jaundice, and biliary pancreatitis) by clinical examination and blood work (total leukocytic count and liver function test)
- Patients' follow-up in OPC was weekly in the first month and then at 2, 6, and 12 months after surgery
- During the 12 months of follow-up, micromalnutrition was assessed if vitamin D was less than 30 g/ml, and macromalnutrition was assessed if hemoglobin was less than 10 g/dl or albumin was less than 3.5 g/dl

## 3. Results

Patients in group 1 were 81.6% females with a mean age of 37.6 years vs. 100% females with a mean age of 40.1 years in group 2, *p* > 0.001. And patients in group 1 had fewer comorbidities and lesser BMI, *p* > 0.001 (Table 2).

Symptomatic GS patients were significantly more in group 1, *p* < 0.001, and the size of the liver by the US was significantly smaller than that in group 2, *p* < 0.001. (Table 3).

**Figure 2:**
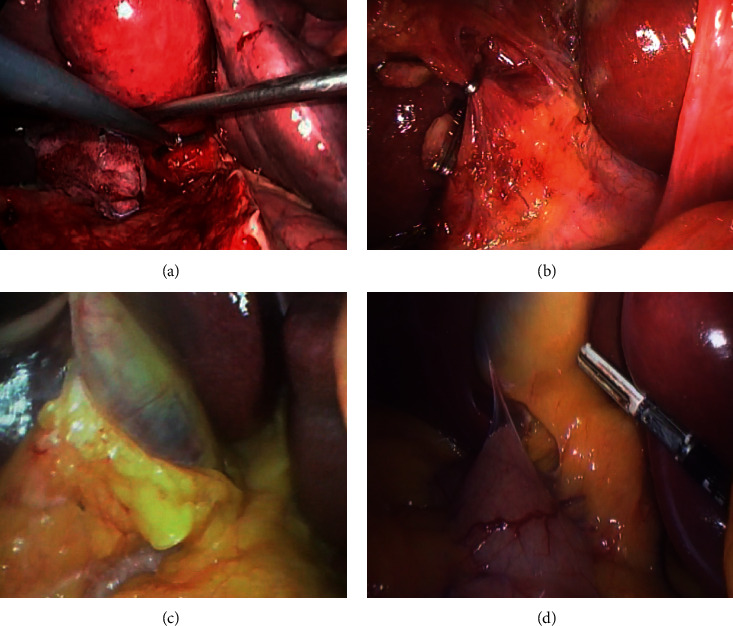
2 patients in group 2 ((a), (c) at time of BS: dense Calot's triangle and adhesion >50%, respectively, and (b), (d) after 2 months).

**Figure 3:**
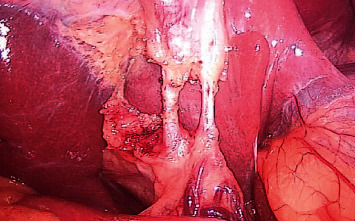
Calot's triangle in delayed cholecystectomy group.

The overall mean LC difficulty score in the concomitant LC vs. delayed LC group was 2.11 ± 0.70 vs. 5.66 ± 0.98, respectively. And also, the overall mean of LC difficulty score in the delayed group during BS vs. after 2 months was 5.66 ± 0.98 vs. 2.26 ± 0.78, respectively, with significant improvement of adhesion at Calot's triangle and to GB, *p* < 0.001 (Tables 4 and 5) (Figures 2 and 3).

Operative time was significantly longer in group 1, *p* < 0.001, with no biliary injury or bariatric surgery complications in both groups. There were 2 cases of postoperative calcular obstructive jaundice, one patient's post LC + LSG in group 1 and one patient after OAGB/MGB in group 2, *p* > 0.001, and both of them were treated conservatively.

Micromalnutrition was higher in group 2, *p* > 0.001, and there was one patient who had macromalnutrition after OAGB/MGB in group 1, *p* > 0.001, but all patients improved at the end of follow-up with nutritional support and vitamins supplements. Moreover, postmeal dyspepsia was significantly higher in group 2, *p* < 0.001 (Table 6).

In the delayed LC group, 3 out of 9 patients (33%) with asymptomatic GS developed symptoms and symptomatic GS patients increased from 25% before BS to 50% within 2 months after BS (*p* > 0.001) (Table 7).

The operative time was significantly longer in group 1 when we had been initially performed BS and GB bleeding was significantly higher, *p* < 0.001 (Table 8).

BMI and EWL % were significantly decreased in both groups, and there was no significant difference between studied groups at different timing of follow-up (Table 9).

## 4. Discussion

Obesity is a major risk factor for the emergence of GS, which is found in 22.8–43.6 % of the morbidly obese undergoing BS. Defining the status of the gallbladder at BS and the degree of any difficulty will facilitate more standardized pathways and management of risk-adjusted outcomes [9, 10].

In this present study, we scheduled the delayed LC to be performed after 2 months from BS to have the benefit of loss of intra-abdominal fat and to avoid any biliary possible complications. We found that the LC difficulty score in group 2 significantly decreased after 2 months from 5.66 ± 0.98 during BS to 2.26 ± 0.78, and this was explained by the easier liver elevation, decreased adhesion at Calot's triangle, and adhesions to GB and decreased BMI, *p* < 0.001.

Scott et al. reported that an advantage of delayed LC is that the procedure might be technically easier to perform as a consequence of the reduced intra-abdominal fat and liver size [11].

While Tustumi et al. reported that the risk for postoperative complications was lower when performed concomitantly LC with BS compared to LC after BS. In addition, the risk for reoperation was lower for concomitant LC. Probably, this is because LC after BS had higher risks as 36.2% of the LC indications following BS were acute cholecystitis or involved common bile duct exploration [12].

In our study, we reported that in (delayed LC group 2) 3 of 9 patients, 33% with asymptomatic GS developed symptoms, and symptomatic GS patients increased from 25% before BS to 50% within 2 months after BS (*p* > 0.001), and although this high ratio is statistically insignificant, it may be attributed due to the limited number of patients in group 2.

In Yardimci et al.'s study of 24 patients who were asymptomatic for GS, and 3 symptomatic patients who underwent LC during SG, there were no complications associated with LC or SG for these patients, and they reported that 79.2% of the patients with GS remained asymptomatic after LSG despite significant weight reduction and five patients (20.8%) experienced biliary colic [4]. Characteristics of these patients who developed symptomatic gallbladder disease (*n* = 5) were not significantly different from those of patients who remained asymptomatic (*n* = 19) [4]. Raziel et al. reported 4 of 43 patients (9.3%) [13] and Sioka et al. reported 3 of 23 (13%) patients who became symptomatic and required LC after LSG [14].

Morais et al. reported that patients who developed symptomatic GS tend to have a higher percentage of excess weight loss (% EWL) [8]. The follow-up period is longer in Yardimci et al.'s study so that they could observe that 20.8% of the patients with asymptomatic GS became symptomatic in the 27-month follow-up period. They believed that the ratio of LC requirement may be higher as the follow-up time lengthens [4].

Some surgeons reported that complications increased during LSG resulting from adding LC: Dakour-Aridi et al. studied 21,137 patients who underwent LSG and were reported in NSQIP, of whom 2.0% underwent concomitant LC. Operative time was increased by 33 minutes, but there was no significant increase in mortality or the number of total adverse events associated with concomitant LC. When complications were individually compared, patients who underwent concomitant LC had higher rates of pneumonia and bleeding. The reason for performing LC was not stated in the NSQIP database [15].

We did not report any cases of postoperative pneumonia, deep venous thrombosis, bleeding, or mortality during our study. Moreover, the incidence of postoperative complications whether biliary or bariatric complications was statistically insignificant between both groups (*p* > 0.05).

On the other hand, we found that operative time significantly increased in group 1 when compared to group 2 with a mean of 92.63 ± 28.25 vs. 68.33 ± 17.49 minutes, respectively (*p* < 0.001). Also, in the concomitant LC, the operative time was significantly longer when LC was done after BS, *p* < 0.001; also, GB bleeding was significantly higher, *p* < 0.001.

Coşkun et al. reported that LC resulted in an additional mean operative time of 49.1 ± 27.9 minutes without any specific complication. There was no statistical difference concerning the overall morbidity (*p* > 0.001) between groups [16].

Tarantino et al. recommended performing LC at the beginning of the operation when the level of patience was still high and the surgeon was not exhausted by the highly demanding BS [17].

Moreover, Leyva-Alvizo et al. recommended planning LC as the initial stage followed by the BS understanding that reconstruction of a common bile duct injury incurs a significantly greater technical challenge and risk [18]. Also, they suggested that in asymptomatic patients, they consider performing the BS initially followed by LC. In addition, they recommended if there was a technically challenging BS or LC procedure, the LC should be postponed to a later date, and it should be part of the consent discussion. They also reported that in minimally symptomatic patients, either delayed or concomitant LC is acceptable, based on patient symptoms and time to elective bariatric surgery, because of the observed low long-term morbidity in several studies as well as acceptable operative time without increasing the length of hospital stay [18].

Dakour-Aridi et al. [15] added 33 minutes for LC and Wood et al. [19] added 27 and 28 minutes during LSG and OAGB/MGB, respectively. In our study, the mean operative time added for LC was 24 minutes which was less, and this may be explained by the selection of technically easier LC according to LC difficulty score.

Our study showed that the incidence of intraoperative GB bleeding was higher in group 1 than that in group 2 (15.8% vs. 8.3%, *p* > 0.001), and there were no biliary injury or bariatric complications in both groups.

During the follow-up period in this study, there were 2 cases of calcular obstructive jaundice: one patient after LSG in group 1 and one patient after MGB in group 2, *p* > 0.001; both of them were treated conservatively via intravenous antibiotics and antispasmodic without the need for ERCP or surgical intervention.

Also, we reported a higher incidence of postmeal dyspepsia in group 2 patients (33.3%) vs. 10.5% in group 1 (*p* < 0.001), but this did not significantly affect patients' micro- or macronutritional status (*p* > 0.001).

Kraag et al. reported in a meta-analysis of controlled studies of GS and dyspepsia a strong association between unspecified food intolerance and gallstones [20].

Wood et al.'s analysis of the 2015 MBSAQIP registry showed that of 98,292 sleeve operations, 2% had concomitant LC. Of 44,427 bypass operations, 3% had concomitant LC. Both LSG and bypass groups had no biliary or BS complication but concomitant LC in the LSG group was associated with increased port site infection (1% versus 4%) in univariate models [19]. In this present study, we have reported port site infection in one patient of LSG in the concomitant group (2.6% vs. 0.0%, *p* > 0.001).

## 5. Conclusion

Patients who will undergo BS with GS disease should be treated differently from the normal population, as we suggest applying a highly selective approach depending on an objective measure of patients' symptoms and operative difficulty, so the time of LC is optimized, and patients are informed during the consent process.

## Figures and Tables

**Table 1:** LC difficulty score.

LC difficulty		Score
Access	BMI ≥45	1
	Difficult liver elevation (liver size ≥ 20 cm at MCL by US)	1

Appearance	<50% of gall bladder (GB)	1
	>50% of GB	2
	Buried GB	3

Distention or contraction	Distended GB	1
	Intrahepatic or contracted GB	1
	Severely inflamed gall bladder (GB): mucocele, pyocele, or thick edematous wall.	2

Dense adhesions at Calot's triangle		1
Total		10

**Table 2:** Demographic data, comorbidities, and anthropometric data.

	Concomitant cholecystectomy group 1 (*N* = 38)		Delayed cholecystectomy group 2 (*N* = 12)		*χ* ^2^/*t*	*p* value
	No.	%	No.	%		
*Demographic data*						
Sex						
Male	7	18.4	0	0.0	2.57	>0.001
Female	31	81.6	12	100.0		

Age (years)						
Mean ± SD	37.65 ± 10.15		40.16 ± 10.27		0.765	>0.001

*Comorbidities*						
Hypertension	7	18.4	3	25.0	0.24	>0.001
DM	4	10.5	3	25.0	1.58	>0.001
Hyperlipidemia	11	28.9	6	50.0	1.85	>0.001

*Anthropometric data*						
BMI (kg/ht^2^)						
Mean ± SD	48.21 ± 8.57		50.51 ± 6.21		0.855	>0.001

*Excess weight (in kg)*						
Mean ± SD	61.82 ± 20.41		66.21 ± 14.78		0.63	>0.001

**Table 3:** Clinical data.

*‡*Clinical data	Group 1 (concomitant cholecystectomy) (*N* = 38)		Group 2 (delayed cholecystectomy) (*N* = 12)		*t*/*X*^2^	*p* value
	No.	%	No.	%		
Biliary symptoms						
Symptomatic	36	94.7	3	25.0	25.84	≤0.001^*∗∗*^
Asymptomatic	2	5.3	9	75.0		

Size of the liver by US (cm in MCL)						
Mean ± SD	17.01 ± 1.59		21.54 ± 1.58		8.54	≤0.001^*∗∗*^

*χ* ^2:^ *chi-square test*; t: *t*-test; ^*∗∗*^*p* value is significant.

**Table 4:** Intraoperative LC difficulty score of the studied patients.

		Group 1 (concomitant cholecystectomy) (*N* = 38)		Group 2 (delayed cholecystectomy) (*N* = 12)		*X* ^2^/*t*	*p* value
		*N*	%	*N*	%		
Level of difficulty							
Access	BMI ≥45 = 1	22	57.9	10	83.3	2.56	>0.001
	Difficult liver elevation (liver size ≥ 20 cm at MCL by US) = 1	3	7.9	10	83.3	26.97	≤0.001^*∗∗*^

Appearance of GB	Adhesion <50%to gall bladder = 1	24	63.2	0	0.0	11.58	≤0.001^*∗∗*^
	Adhesion >50% to gall bladder = 2	8	21.1	12	100.0	23.68	≤0.001^*∗∗*^
	Completely buried GB = 3	0	0.0	0	0.0	0.0	>0.001

Acutely inflamed (thick edematous wall, pyocele, or mucocele = 2		1	2.6	2	16.7	3.18	>0.001
Distended GB = 1		0	0.0	5	41.7	17.59	≤0.001^∗∗^
Contracted or intrahepatic GB = 1		0	0.0	3	25.0	10.11	≤0.001^*∗∗*^
Dense adhesion at Calot's triangle = 1		9	23.7	12	100.0	21.8	≤0.001^*∗∗*^
*Total difficulty score (10)*							
Mean		2.11 ± 0.70		5.66 ± 0.98		10.32	≤0.001^*∗∗*^

^*∗∗*^p value is significant.

**Table 5:** Intraoperative difficulty in delayed cholecystectomy group (group 2) before and after 2 months of bariatric surgery.

		During BS		After 2 months		*p* value
Level of difficulty		No	%	No	%	
Access	BMI ≥45 = 1	10	83.3	2	16.7	≤0.001^*∗∗*^
	Difficult liver elevation (liver size ≥ 20 cm at MCL by US) = 1	10	83.3	9	75.0	>0.001

Appearance of GB	Adhesion to GB <50% = 1	0	0.0	9	75.0	≤0.001^*∗∗*^
	Adhesion to GB>50% = 2	12	100.0	0	0.0	≤0.001^*∗∗*^
	Completely buried GB = 3	0	0.0	0	0.0	>0.001

Acutely inflamed (thick edematous wall, pyocele, or mucocele = 2		2	16.7	1	8.3	>0.001
Distended GB = 1		5	41.7	0	0.0	≤0.001^*∗∗*^
Contracted or intrahepatic GB = 1		3	25.0	3	25.0	>0.001
Dense adhesion at Calot's triangle = 1		12	100.0	3	25.0	≤0.001^*∗∗*^
*Total difficulty score(10)*						
Mean		5.66 ± 0.98		2.26 ± 0.78		≤0.001^*∗∗*^

McNemar test and paired *t*-test. ^*∗∗*^p value is significant.

**Table 6:** Type of bariatric surgery, operative details, and complications.

	Group 1 (concomitant cholecystectomy) (*N* = 38)		Group 2 (delayed cholecystectomy) (*N* = 12)		*t*/*X*^2^	*p*
	No	%	No	%		
*Type of operation*						
LSG (*n* = 44)	35	92.1	9	75.0	2.52	>0.001
OAGB/MGB (*n* = 6)	3	7.9	3	25.0		

*Operative time of BS*						
Operative time (min)						
Mean ± SD	92.63 ± 28.25		68.33 ± 17.49		2.83	≤0.001^*∗*^

*Intraoperative complications*						
GB bleeding	6	15.8	1	8.3	0.42	>0.001
Biliary injury	0	0.0	0	0.0	0.0	>0.001

*Postoperative complications*						
Calcular obstructive jaundice	1	2.6	1	8.3	2.02	>0.001
Port site infection	1	2.6	0	0.0	0.98	>0.001
Bleeding	0	0.0	0	0.0	0.0	>0.001
Leakage	0	0.0	0	0.0	0.0	>0.001

*Nutritional complications*						
Micromalnutrition	7	18.4	3	25.0	1.14	>0.001
Macromalnutrition	1	2.6	0	0.0	0.89	>0.001
Postmeal dyspepsia	4	10.5	4	33.3	12.3	≤0.001^*∗∗*^

^*∗*^, ^*∗∗*^*p* value is significant.

**Table 7:** Incidence of biliary symptoms in group 2 before and after BS.

*N* = 12	Before bariatric surgery		After bariatric surgery (2ms)		MaNemar	*p* value
	*N*	%	*N*	%		
Symptomatic	3	25.0	6	50.0	2.54	≤0.001
Asymptomatic	9	75.0	6	50.0		

**Table 8:** Intraoperative data among the studied concomitant cholecystectomy patients in relation to timing before or after bariatric surgery.

	Pre-bariatric surgery subgroup A (*N* = 31)	Post-bariatric surgery subgroup B (*N* = 7)	*t*-test/*χ*^2^	*p* value
*Operative time (min)*				
Mean ± SD	84.19 ± 19.62	130.0 ± 31.62	4.756	≤0.001^*∗∗*^

*Total difficulty score*				
Mean	2.11 ± 0.741	2.1 ± 0.688	0.04	>0.001

GB bleeding	2 (6.5%)	4 (57.1%)	11.3	≤0.001^*∗∗*^

^*∗∗*^ *p* value <0.05 is significant.

**Table 9:** EWL at 12 months and BMI change after operation among the studied groups.

BMI	Concomitant cholecystectomy (*N* = 38)	Delayed cholecystectomy (*N* = 12)	*t*	*p* value
*BMI: 3 months*				
Mean ± SD	40.72 ± 7.44	39.33 ± 5.83	0.59	>0.001

*BMI: 6 months*				
Mean ± SD	36.95 ± 6.87	35.25 ± 4.82	0.79	>0.001

*BMI: 9 months*				
Mean ± SD	33.18 ± 5.9	31.75 ± 3.91	0.78	>0.001

*BMI: 12 months*				
Mean ± SD	30.84 ± 4.79	29.25 ± 3.25	1.07	>0.001

*EWL/12 (%)*				
Mean ± SD	78.04 ± 16.6	84.58 ± 8.45	1.304	>0.001

## Data Availability

Data used to support the findings of this study are available from the corresponding author upon request.
